# Modern Wasp Waists

**Published:** 1892-12

**Authors:** 


### MODERN WASP WAISTS.

A study of the matter of proportion in the human figure during the last fifteen years, led us long ago to the belief that the average civilized woman, at least in this country, has a waist too small for the rest of her body. We will not venture to say, as some irascible critics have said, that the narrow waists of our American women are productive of waspishness of temper, but we have demonstrated by a very careful study of the matter in hundreds of cases that smallness of waist in woman is a deformity, and is invariably connected with other deformities, such as prolapsus of the stomach and bowels, dilatation of the stomach, prolapsus of one or both kidneys, displacement of the womb, congestion and displacement of the ovaries, and other morbid conditions which grow out of those mentioned. It is a small waist which renders the American woman so much of an invalid, and so feeble, when compared with man, that she has come to appropriate without remonstrance the appellation, " the weaker vessel." What is the proper waist proportion ? is a question which has been earnestly asked by those who are seeking through physical culture and the adoption of a healthful mode of dress, to acquire natural symmetry and proportion.

We have endeavored to solve this question by the study of the waists of peasant women and the women of savage or semi-savage tribes, and lastly by a careful study of the proportion of ancient statues of women. We made the basis of our study the relation of the waist measure to the height of the individual. This proportion is obtained by dividing the waist measure by the height, the result being the percentage of the height represented by the waist. In the Venus de Milo we find the waist to be 47.6 per cent. of the height, while in the average American woman it is less than 40 per cent. of the height, which means a difference of several inches. A woman five feet four inches in height, with the same proportions as the Venus de Milo, would have a waist measure of 30 inches. A waist 40 per cent. of the height would be but 25.6 inches. The difference between these two measurements must show just the difference between robustness and abundant vitality, and weakness, suffering, disease, and inefficiency.—*Good Health.*

